# Mechanisms of Copper-Induced Autophagy and Links with Human Diseases

**DOI:** 10.3390/ph18010099

**Published:** 2025-01-15

**Authors:** Yuanyuan Fu, Shuyan Zeng, Zhenlin Wang, Huiting Huang, Xin Zhao, Min Li

**Affiliations:** 1 Guangzhou Municipal and Guangdong Provincial Key Laboratory of Molecular Target & Clinical Pharmacology, The NMPA and the State Key Laboratory of Respiratory Disease, School of Pharmaceutical Sciences, Guangzhou Medical University, Guangzhou 511436, China; 2 School of Pharmaceutical Sciences, Sun Yat-Sen University, Guangzhou 510006, China

**Keywords:** autophagy, copper, copper-based agents, human diseases, therapeutic potential

## Abstract

As a structural and catalytic cofactor, copper is involved in many biological pathways and is required for the biochemistry of all living organisms. However, excess intracellular copper can induce cell death due to its potential to catalyze the generation of reactive oxygen species, thus copper homeostasis is strictly regulated. And the deficiency or accumulation of intracellular copper is connected with various pathological conditions. Since the success of platinum-based compounds in the clinical treatment of various types of neoplasias, metal-based drugs have shown encouraging perspectives for drug development. Compared to platinum, copper is an essential intracellular trace element that may have better prospects for drug development than platinum. Recently, the potential therapeutic role of copper-induced autophagy in chronic diseases such as Parkinson’s, Wilson’s, and cardiovascular disease has already been demonstrated. In brief, copper ions, numerous copper complexes, and copper-based nano-preparations could induce autophagy, a lysosome-dependent process that plays an important role in various human diseases. In this review, we not only focus on the current advances in elucidating the mechanisms of copper or copper-based compounds/preparations on the regulation of autophagy but also outline the association between copper-induced autophagy and human diseases.

## 1. Introduction

Autophagy is a highly conserved intracellular degradation system that transports intracellular cargo to the lysosome for degradation and recycling [1]. It is well known that autophagy regulates the energy balance of individual cells and maintains the overall balance of the organism’s metabolism [2]. Autophagy dysfunction has been implicated in the pathophysiology of various human diseases, including cancer, neurodegeneration diseases, cardiovascular diseases, and various metabolic disorders, especially cancer [3]. Although the role of autophagy in cancer is controversial, there is increasing evidence indicating that autophagy ensures cancer cells survive under stress and increases resistance to radiation and chemotherapy in established cancers at the later stages [4,5]. These observations have led to increasing concerns about using autophagy inhibitors as potential antitumor agents. Therefore, autophagy may be a potential target for therapeutic drug development in human diseases.

Metals have unique biological properties in living organisms, and the transition metal copper has advantages over platinum compounds as an endogenous substance [6]. Copper is a structural and catalytic cofactor that plays a critical role in various biological pathways, including mitochondrial respiration, antioxidant defense, and neurotransmitter synthesis, and is intimately involved in cell proliferation and death [7,8,9,10,11]. Due to the ability of copper to directly damage different intracellular components or disrupt redox balance, the accumulation of copper can be highly toxic [12,13]. So, copper homeostasis is tightly regulated. And understanding the mechanisms that promote or counteract copper toxicity is important because it has the potential to expose potential therapeutic targets for human diseases. In recent years, copper has been shown to trigger autophagy, a lysosome-dependent degradation process that plays an important role in regulating the survival of cells under various stressful conditions [14]. Therapeutically, copper-based compounds are potential therapeutic agents that can be used to treat human disease, particularly cancer. A recent study has found that copper complexes can induce autophagy to cause cancer cell death [15]. In addition, copper-based nano-preparations developed using various unique technologies have been shown to cause cancer cell death by inducing autophagy [16]. In fact, copper-based agents also fight drug resistance in cancer cells [17,18]. Therefore, further understanding the signaling of copper-induced autophagy is significant for the development of novel anticancer strategies and solving the problem of resistance. Additionally, a recent study confirmed that occupational copper exposure increases the risk of developing Parkinson’s disease (PD) in close association with copper-activated autophagy [19]. Moreover, it has also been shown that copper-induced autophagy may be a potential therapeutic target for other human diseases, e.g., Wilson’s disease (WD) and cardiovascular disease [20,21,22,23].

Herein, we summarize the specific mechanisms and characteristics of copper-induced autophagy and focus on the relationship between copper-induced autophagy and human diseases, including cancer, PD, WD, and other disorders. In addition, we focus on the effects of copper ions, copper complexes, and copper-based nano-preparations on cancer cell survival, angiogenesis, cancer treatment efficacy, and drug resistance to elucidate the potential of autophagy-induced copper-based formulations as therapeutic agents for human diseases.

## 2. Effects of Copper on Autophagy

It is interesting to note that several recent studies have shown that the transition metal copper can induce autophagy. Copper is a transition metal with unique properties, such as redox and catalytic properties, variations in the number of ligands, and inherent properties of cationic metal ions and ligands [24]. Consequently, copper can exist not only in ionic form but also combine with various ligands to form different types of complexes and even copper-centered construction of nanomaterials and nanoplatforms.

### 2.1. Copper Ions

**Figure 1:**
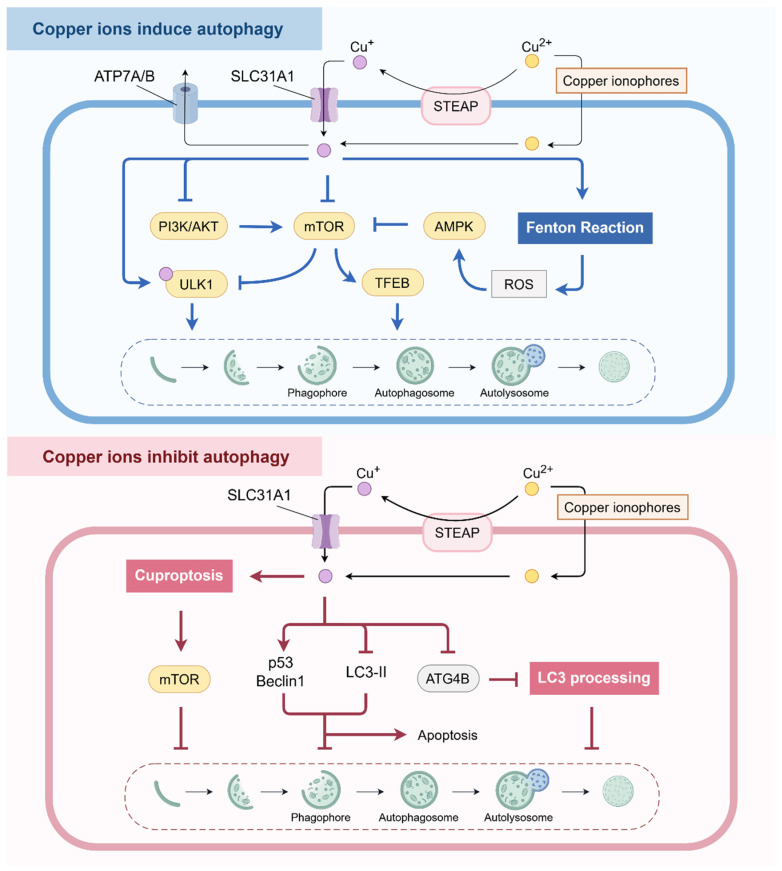
**The effect of copper ions on autophagy and the mechanisms involved.** Cu^+^ enters the cytosol through SLC31A1. Cu^2+^ enters the cytoplasm directly through copper ionophores or is reduced to Cu^+^ by STEAP. Copper can induce autophagy either by directly activating ULK1 or via the PI3K/AKT/mTOR pathway to activate ULK1. Moreover, copper generates ROS via the Fenton reaction, thus inducing autophagy by stimulating the AMPK/mTOR pathway. Copper is capable of directly initiating autophagy by inhibiting mTOR to stimulate TEFB. Conversely, copper can induce cuproptosis to increase the phosphorylation of mTOR, thereby inhibiting autophagy. In addition, copper inhibits ATG4B to hinder LC3 processing, which inhibits autophagy. Copper can also inhibit autophagy and induce apoptosis through the up-regulation of Beclin-1 and p53 proteins and the down-regulation of LC3-II protein. The red and blue solid lines indicate the different effects of copper ions on autophagy.

Copper is a vital trace metal element, which exhibits strong redox properties and is present in the body in the forms of Cu^+^ and Cu^2+^ [25]. Cu^+^ is unstable both in vivo and in vitro and is readily converted to the more stable Cu^2+^ [26]. Heavy metal-induced cell death such as copper is mainly associated with reactive oxygen species (ROS) generated by Fenton or Fenton-like reactions [27]. It is well documented that autophagy plays a protective role in cells, mitigating the harmful effects of oxidative stress and thus preventing damage to the cell [28,29], and this maintains the homeostatic balance within the cell. Here, we summarize the effects of copper ions on autophagy and the mechanisms involved (Figure 1).

#### 2.1.1. Copper Ions Induce Autophagy

Copper has been reported to interact directly with autophagic kinase ULK1 [unc-51-like autophagy activating kinase 1] and ULK2 [unc-51-like autophagy activating kinase 2] to activate autophagy [30]. A recent study showed that excess Cu^2+^ led to a reduction in the level of the autophagy inhibitor mechanistic target of rapamycin (mTOR), which subsequently increased the level of Beclin-1 [31]. A further investigation revealed that Cu^2+^ could stimulate ULK1 activity and enhance Beclin-1 expression, thus initiating autophagy through the suppression of mTOR signaling [32]. In addition, Cu^2+^-induced oxidative stress was found to be capable of inducing the formation of autophagic vesicles, as evidenced by a decrease in mTOR gene expression and an increase in Atg5 gene expression [33]. Moreover, the release of Cu^+^ from Cu_2_O crystals has been shown to cause autophagic death of endothelial cells through the generation of ROS and the subsequent activation of AMPK via superoxide [34]. Further studies have shown that Cu_2_O crystals can lead to the degradation of p62, the processing of LC3, and the elevation of LC3 puncta [34], suggesting the activation of autophagy.

In addition, the PI3K/Akt pathway has the capacity to exert a regulatory effect on autophagy, whereby it facilitates the phosphorylation of mTOR [35]. It has been shown that Cu^2+^, present in CuSO_4_, induces autophagy through the induction of oxidative stress and subsequent stimulation of the PI3K/AKT/mTOR pathway [36]. The principal findings were that CuSO_4_ treatment resulted in a reduction in the mRNA levels of TORC1, TORC2, and LC3-I, and an increase in the mRNA levels of PI3K, AKT1, Beclin-1, Atg4B, LC3-II, and Atg5 [36]. Likewise, the mRNA levels and protein expression of autophagy markers (PI3K, Akt, Atg5, LC3, Atg4B, and Beclin-1) were found to be elevated in brain tissues following 12 weeks of treatment with 300 mg/kg of copper sulfate (CuSO_4_) [37]. However, no significant change was observed in mTORC at this time [37]. At higher copper concentrations, the level of mTORC was found to be down-regulated, indicating that excess copper induces autophagy through the activation of the PI3K/Akt/mTOR pathway [37]. It has been demonstrated that chronic copper exposure results in copper toxicity and AMPK phosphorylation-mediated autophagy activation [19]. Interestingly, CuSO_4_ was found to induce autophagy in cells via the ROS-AMPK-mTOR pathway [38]. Additionally, it has been found that in ATPase Copper Transporting Beta (ATP7B)-deficient cells, copper accumulation is caused by the absence of the copper efflux transporter [20]. Consequently, activation of mTORC1 was inhibited and TFEB was activated in ATP7B-KO cells, thus favoring a stimulation of autophagy [20].

#### 2.1.2. Copper Ions Inhibit Autophagy

Copper overload has been demonstrated to induce cell death, including apoptosis, ferroptosis, and cuproptosis [14]. The effects and mechanisms of copper on cell death have been well reviewed by scholars and will not be elaborated upon here [14]. Nevertheless, there appears to be a robust association between copper ion-induced cell death and its inhibition of autophagy. Copper ions have been demonstrated to induce an increase in dihydrolipoamide S-acetyltransferase (DLAT) through the process of cuproptosis [17]. Subsequently, copper ions with inducing cuproptosis increase phosphorylation of the mechanistic target of rapamycin (mTOR), which inhibits autophagy [17]. Cu^2+^-containing lactoferrin hydrolysate (LFH) mixtures have been shown to activate apoptosis and inhibit autophagy by potentially cross-talking the caspase-3 and p53 pathways through the up-regulation of Beclin-1 and p53 proteins and the down-regulation of LC3-II protein [39]. Likewise, our previous study found that copper inhibits autophagy [21]. However, it is not associated with the mechanism of cell death induced by copper ions. We found that copper inhibits autophagy by directly disrupting the protein structure of ATG4B to inhibit protease activity and promotes the formation of p62 insoluble aggregates, oligomeric and aggregated states of ATG4B [21].

Overall, the present findings demonstrate that copper ions can activate autophagy mainly by regulating the upstream signaling pathways such as the PI3K/AKT/mTOR pathway. However, studies have shown that copper ions can also inhibit autophagy. This opposite effect of copper on autophagy may be because the current research on the regulation of autophagy by copper is not sufficiently advanced. Therefore, more in-depth studies are also needed to explore the exact mechanisms by which copper ions regulate autophagy and to illustrate the application of copper-based agents for treating human diseases.

### 2.2. Copper Complexes

**Figure 2:**
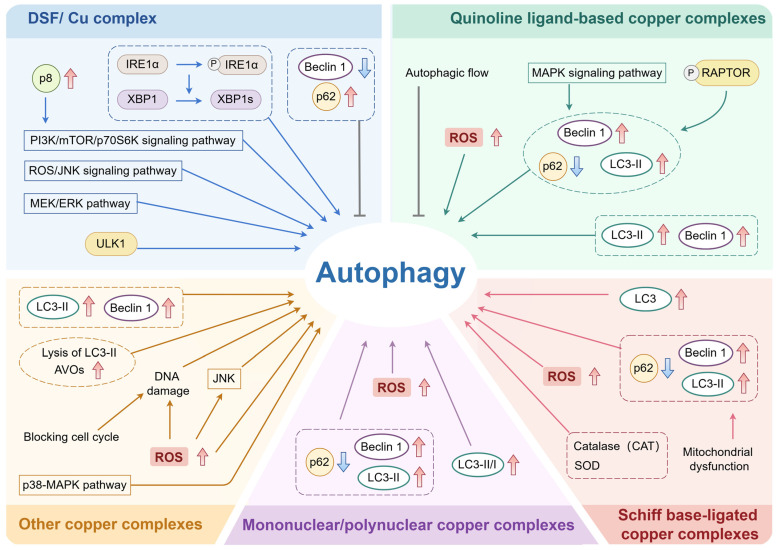
**Induction of autophagy by multiple copper complexes and diverse mechanisms involved.** A variety of copper complexes can have different influences on autophagy through different mechanisms and are classified according to their ligands as the DSF/copper complex, quinoline ligand-based copper complexes, Schiff base-ligated copper complexes, mononuclear/polynuclear copper complexes, and other copper complexes.

Compared with copper ions, copper complexes and copper-based nanoparticles (NPs) have diverse linkage pathways into the cell membrane. For example, Peng et al. monitored the delivery and release of doxorubicin–copper complexes in cells using fluorescence lifetime imaging microscopy, which found the process to be like the pro-drug process [40]. In addition, the complex bis-diethyldithiocarbamate–copper (DDC-Cu) obtained by the reaction of DSF with copper ions can penetrate the cancer cell membrane and enter the cell [41]. In fact, the physicochemical properties of copper complexes themselves can confer the ability to cross cell membranes [42]. Therefore, copper-containing preparations are promising therapeutic agents because of their ability to subtly enter cells and act through copper ions or other reactions. In addition to the induction of autophagy by copper ions, copper complexes subtly coordinate autophagic activity, and there are similarities but also differences in the mechanisms by which copper ions and copper complexes induce autophagy. Currently, the research on the regulation of autophagy by copper complexes is predominantly focused on copper (II) complexes, and there are few studies on copper (I) complexes [43,44]. Here, we summarize the effects of diverse copper complexes on autophagy and the related mechanisms involved (Figure 2).

#### 2.2.1. Disulfiram (DSF)/Copper Complex

DSF is a well-known anti-alcoholic drug, and studies in the field of drug repurposing have found that DSF forms a potent complex with copper (DSF/Cu) that induces apoptosis [45]. Interestingly, a growing number of studies have found that DSF/Cu is also capable of regulating autophagy, including both activation and inhibition of autophagy. For example, the DSF/copper complex can induce cellular autophagy activation by directly targeting ULK1 [46]. Zhang et al. have confirmed that DSF/Cu induces autophagy activation in cancer stem cells through sustained activation of the MEK/ERK pathway [47]. And Guo et al. demonstrated that the DSF/copper complex induces autophagy by activating the ROS/JNK signaling pathway [48]. In addition, the transcription factor p8 was shown to play a key role in the cellular stress response and was involved in DSF/Cu treatment-induced autophagy in pancreatic cancer cells [15]. Specifically speaking, DSF/Cu could stimulate autophagy by inducing high p8 expression in pancreatic cancer cells, consequently stimulating autophagy by the p8-mediated PI3K/mTOR/p70S6K signaling pathway [15]. Furthermore, DSF/Cu can induce autophagy activation by directly or indirectly activating UPR sensor IRE1α, thereby promoting the phosphorylation of IRE1α and its downstream XBP1 splicing into active XBP1s [45]. However, Zha et al. found that DSF/copper significantly inhibits autophagy in MOLM-13 cells, characterized by a reduction in the level of the pro-autophagic protein Beclin-1 and enrichment of the abundance of p62 [49].

#### 2.2.2. Quinoline Ligand-Based Copper Complexes

Actually, quinoline and its derivatives are often chosen as ligands to coordinate with metal ions to form metal complexes [50,51,52]. One study showed that quinoline copper (II) complexes inhibited autophagy by blocking autophagic flow [50]. Another study found that copper (II) complexes of haloquinoline Schiff base derivatives inhibited autophagy by hindering the fusion of autophagosomes with lysosomes, enhancing autophagosomes, and inhibiting autophagic fluxes [53]. However, Cu (II) complexes based on quinoline-substituted tripyridine ligands [Cu(4′-(2-quin)-terpy) Cl] (PF_6_) have been shown to activate autophagy by inducing intracellular ROS [54].

Similarly, isoquinoline derivatives can be used as ligands to form well-activated complexes with copper [55]. A study showed that copper (II) complexes with isoquinoline derivatives as ligands could induce autophagy through activation of the MAPK signaling pathway, characterized by up-regulation of LC3-II and Beclin-1 and down-regulation of p62 and LC3-I expression [56]. In addition, a recent study found that the copper (II) indene–isoquinoline complex WN197 activated autophagy by causing phosphorylation of RAPTOR in the mTOR complex, leading to Beclin-1 accumulation, LC3-II formation, and p62 degradation [57]. Similarly, a novel copper indene–isoquinoline derivative, WN198, has been reported to induce autophagy in cancer cells, as evidenced by Beclin-1 accumulation and LC3-II formation [58].

#### 2.2.3. Schiff Base-Ligated Copper Complexes

Over the past few decades, Schiff bases and their complexes have become widely known for their wide range of biological potential [59]. The Schiff base Cu (II) complex has been shown to induce autophagy in MCF-7 breast cancer cells, producing the autophagy marker LC3 protein after 72 h of treatment with the copper complex [60]. The copper (II) complex Cu (Cl_2_-L^1^) Cl with a tridentate halogen-substituted Schiff base ligand can equally trigger autophagy to promote cell death [61]. Moreover, the mechanistic study suggested that copper complexes based on the salicylaldehyde Schiff base L^1–9^H_2_ ligand can induce A549/DDP autophagy via the mitochondrial dysfunction pathway, characterized by up-regulation of the expression levels of Beclin-1 and LC3-II/I and down-regulation of p62 [62]. Furthermore, Xia et al. have proved that increased ROS production caused by the Schiff alkali copper coordination compound (SBCCC) could activate autophagy [63]. Interestingly, other research has shown that the Cu (II) complex of Schiff base [Cu (DAAUPicH-1) Br]_2_ could activate autophagy via superoxide dismutase (SOD) and catalase (CAT) [64].

#### 2.2.4. Mononuclear/Polynuclear Copper Complexes

In recent years, a number of mononuclear and multinuclear copper (II) complexes have been synthesized [65]. Wang et al. have synthesized and verified that novel mono-, bi-, tri-, and tetra-nuclear copper complexes could induce autophagy activation by significantly increasing the expression levels of Beclin 1 and LC3-II while decreasing the expression level of p62 in HUVECs [66]. Moreover, the dinuclear copper (II) complex has been shown to activate autophagy in A2780 cells, leading to cell death [67]. Rosin derivatives have a variety of biological activities and are able to act as ligands to form complexes with metals [68,69]. Novel optically pure dinuclear copper (II) complexes of the rosin derivative dehydroabietic acid (DHA, HL) have been reported to promote the conversion of LC3-I to LC3-II and induce autophagic cytotoxicity in MCF-7 cells [69]. The optically pure chiral dinuclear copper (II) complex [Cu_2_(μ-Cl)_2_L_2_]-CH_2_Cl_2_ could cause cell death by inducing ROS generation to activate autophagy [70,71]. Furthermore, the single-molecular heteropolynuclear Er (III)-Cu (II) complex (ErCu_2_) could induce autophagy activation in tumor cells [72].

#### 2.2.5. Other Copper Complexes

Researchers have synthesized several novel copper complexes that induce autophagy by selecting or modifying different ligands [73,74]. In the first place, bisbridged copper (II) phenoxy complexes and heterocyclic copper (II) complexes have been shown to induce autophagy activation in A2780 cells [75,76]. It has been proved that copper (II) complexes with β-diketone and 1,10-phenanthroline (CBP-01) activate autophagic processes in sarcoma 180 cells [77]. Man et al. found that copper (II) isopropyl 2-pyridone thiosemicarbazone compounds could activate autophagy [43]. Another study reported that troglitazone-containing Cu (II) complexes could induce autophagy and up-regulate Beclin-1 and LC3-II expression in MGC80-3 cells [78]. Identically, ternary copper complex [Cu(phen)(L-tyr)Cl]·3H_2_O could induce autophagy activation by up-regulation of LC3-II expression in MCF-7 and MDA-MB-231 cells [79]. Moreover, copper complexed with benzaldehyde nitrogen mustard-2-pyridine carboxylhydrazone (BNMPH) could induce autophagy in HepG2 cells, as evidenced by an increase in the formation of acidic vesicular organelles (AVOs) and lysis of LC3-II [80]. Particularly, the novel anticancer copper complex (hYF127c/cu) has been reported to activate autophagy by up-regulating autophagy transcription genes through activation of the p38 MAPK pathway [81].

Other reported copper complexes regulate autophagy mainly by affecting ROS. For example, copper (II) complexes of asymmetric halogen-substituted [NNʹO] ligands have been shown to trigger autophagy by inducing an increase in oxidative stress [74]. Copper (II) complexes with 2,2′:6′,2′′-terpyridine could increase intracellular ROS and interfere with cell cycle progression, thus leading to cell death by apoptosis and autophagy [82]. Furthermore, the Cu (II) complex with 4-fluorophenoxyacetic acid hydrazide could induce genomic DNA damage by blocking the cell cycle and promoting ROS generation, thereby activating autophagy and triggering cell death [83]. Trejo-Solís et al. reported that the copper compound Cas III-ia promoted the accumulation of intracellular ROS, leading to the sustained activation of JNK, which in turn induced autophagy activation in C6 glioma cells [84]. Moreover, copper (II) bis (thiosemicarbazone) derivatives could increase ROS production in the p53 wild-type A549 cell line and activate autophagy [85].

### 2.3. Copper-Based NPs

**Figure 3:**
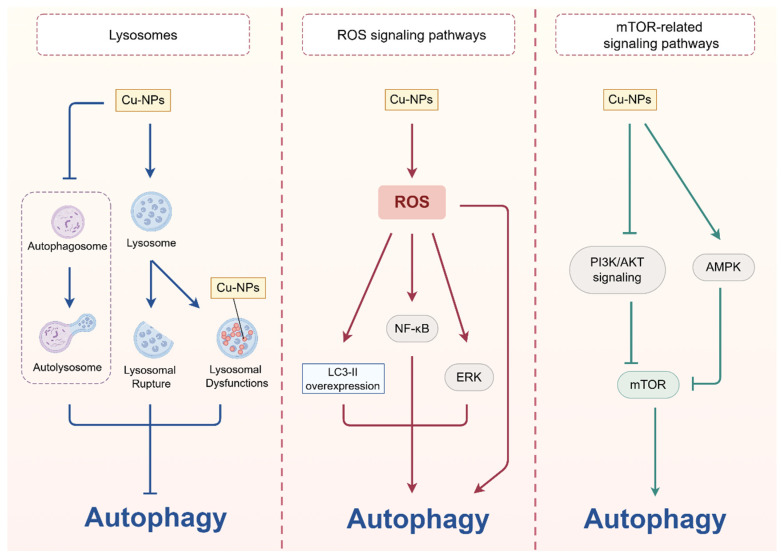
**Mechanisms of autophagy regulation by copper-based NPs.** Copper-based NPs are capable of inducing or inhibiting autophagy in diverse mechanisms. First, copper-based NPs can cause lysosomal dysfunctions or promote lysosomal rupture and also inhibit autophagosome–lysosome fusion, thus inhibiting autophagy. Further, by regulating ROS signaling pathways, copper-based NPs can increase ROS generation to directly induce autophagy or increasing the expression of LC3-II to induce autophagy, or via activating ROS/ERK or the NF-κB pathwayto induce autophagy. In addition to modulating mTOR-related signaling pathways, copper-based nanoparticles stimulate different upstream factors to induce autophagy via the AMPK/mTOR and PI3K/AKT/mTOR signaling pathways.

Recently, copper-based NPs (e.g., Cu/Cu_2_O/CuO NPs) have also been shown to regulate autophagy. For example, phosphatidylferrin-encapsulated copper NPs (AFtCu) can lead to overexpression of the autophagy markers LC3 and p62, thus triggering autophagy [86]. Zhou et al. have developed and demonstrated that nona-copper (II)-containing 18-tungsto-8-arsenate (III) could induce autophagy in HepG2 cells [87]. However, the role of copper-based NPs in the regulation of autophagy is still not clear. Here, we summarize the potential mechanisms and characteristics of copper-centric NPs and nanoplatforms constructed at this stage to regulate autophagy (Figure 3).

#### 2.3.1. Copper-Based NPs Affect the Lysosomes

Lysosomes play an important role in the degradation of dysfunctional proteins, intracellular pathogens, and damaged organelles to maintain cellular homeostasis [88]. Studies indicated that the cellular deposition of NPs can cause lysosomal dysfunctions and thus autophagic stress [89,90]. It has been reported that copper oxide NPs (CuO NPs) are mainly deposited in lysosomes, which damages lysosomes thereby blocking autophagic flux and accumulation of undegraded autophagosomes [16]. Another study showed that PDA-PEG/copper (Poly/Cu) nanocomplexes inhibited the autophagic pathway by promoting lysosomal rupture, manifested by Poly/Cu increasing the expression of LC3B-II [91]. Subsequently, Jia et al. designed human H-ferritin (HFn), regorafenib, and Cu^2+^ into brain-targeted nanoplatforms (HFn-Cu-REGO NPs), which showed that the nanoplatform induced fatal autophagic arrest in GBM cells by inhibiting autophagosome–lysosome fusion [92].

#### 2.3.2. Copper-Based NPs Regulate ROS

ROS also plays an important role in copper-based nanoparticle-induced autophagy, which may be one of the important mechanisms. For example, it has been reported that cuprous oxide NPs (CONPs) can induce autophagy by activating the ROS/ERK signaling pathway in bladder cancer cells [93]. In addition, it has been shown that zinc-doped copper oxide nanocomposites (Zn CuO NPs) can induce autophagy via ROS and cross-talk with activation of the NF-κB pathway [94]. Moreover, Xiong et al. have developed a therapeutic copper complex encapsulated in a natural nanocarrier phosphatidylferrin (AFt-Cu), which has been demonstrated to increase ROS levels in cells, leading to LC3-II overexpression and ultimately induce autophagy in cancer cells [95]. Similarly, near-infrared optically active D-Cu_2−x_S nanocrystals (NCs) could induce significant autophagy enhancement by increasing intracellular ROS production [96]. Furthermore, Song et al. firstly systematically reported that cuprous oxide NPs (Cu_2_O-NPs) adsorbed to serum proteins in cell culture medium were internalized by uveal melanoma cells through lipid raft-mediated endocytosis, thereby localizing in autolysosomes and lysosomes, causing damage, resulting in increased levels of ROS and overactivation of autophagy [97].

#### 2.3.3. Copper-Based NPs Regulate mTOR-Related Signaling Pathways

Many studies have shown that mTOR is a convergence point for copper-based NPs to induce autophagy through multiple signal transduction pathways [98,99]. Kang et al. found that copper NPs could induce autophagy via the PI3K/AKT/mTOR and AMPK/mTOR pathways [98]. Moreover, researchers have proved that nanosized copper particles (nano Cu) could induce autophagy by down-regulating the activation of the Akt/mTOR/p70S6K pathway in mesenchymal cells [100]. In addition, another study showed that copper NPs induced oxidative stress in the rat testes and promoted autophagy via the AKT/mTOR pathway [101].

## 3. Copper-Induced Autophagy and Cancer

Chemotherapy is an effective antitumor treatment that has been used for a long time and has achieved good benefits [102,103,104]. Platinum-based chemotherapeutic agents such as cisplatin, carboplatin, oxaliplatin, etc., have been approved globally for the treatment of human cancers, and whereas they have been more successful in the treatment of cancers, the low selectivity and the occurrence of drug resistance have limited the further dissemination of these compounds [105,106]. Recently, copper compounds have attracted more attention as anticancer agents [24], as it is hypothesized that endogenous metals may be less toxic to normal cells than cancer cells. Therefore, copper-based agents capable of selectively killing tumor cells and combating drug resistance are a good alternative to platinum-based anticancer compounds. Given that the relationship between copper and cancer and the application of copper complexes as anticancer agents have already been well summarized [24,107,108], this review will not go into too much detail. Here, we just summarize copper ions, copper complexes, and copper nanos in the treatment of cancer via regulating autophagy (Table 1).

### 3.1. Copper Ions

It is well known that prostate cancer is the most common malignancy in men worldwide [115]. Docetaxel, a paclitaxel analog, is currently the first-line chemotherapy drug for prostate cancer [116]. However, developing resistance to docetaxel prevents prostate cancer patients from benefiting from it [117]. Elysium copper chloride (ES-CuCl_2_) could enhance the chemosensitivity of docetaxel and inhibit prostate cancer growth through copper ion-induced autophagy [17]. Furthermore, adding Cu^2+^ to digested bovine lactoferrin hydrolysate could enhance the anticancer effect of AGS cells, which was achieved by both activating apoptosis and inhibiting autophagy [39]. These findings suggest that regulating autophagy with copper ions could enhance cancer treatment.

### 3.2. Copper Complexes

#### 3.2.1. Inhibiting Cancer Cell Proliferation and Angiogenesis

Recently, DSF/Cu-activated autophagy has been shown to be involved in its inhibition of proliferation and induction of apoptosis in human pancreatic cancer cells [15]. Further research showed that the DSF/copper complex effectively inhibited the proliferation of colorectal cancer by activating autophagy leading to cell death via the ULK1 pathway [46]. Moreover, the copper (II) complex Cu (Cl_2_-L_1_) Cl with a tridentate halogen-substituted Schiff base ligand could exert antiproliferative activity via autophagy activation in the A2780 ovarian cancer cell line [61]. Furthermore, it has been confirmed that the BNMPH-Cu complex inhibits the proliferation of HepG2 and HCT-116 cells through activation of autophagy, and possesses strong antitumor activity [80]. Another study also showed that DpdtaA-Cu had a promising antiproliferative effect on hepatocellular carcinoma and autophagic lysosomal cell death was involved to some extent in the antiproliferative effect of DpdtaA-Cu [109]. Moreover, Wang et al. have confirmed that the induction of autophagy by the DpdtpA copper complex (DpdtpA-Cu) was significantly stronger than that of DpdtpA, but this was not related to the strength of the antiproliferative activity [118].

Moreover, it has been shown that the autophagy-promoting effect of trinuclear Cu complexes inhibits the growth and proliferation of endothelial cells and effectively suppresses angiogenesis in tumor cells [66]. Additionally, optically pure chiral copper (II) complexes of rosin derivatives [CuL_4_Cl] Cl·2CH_2_Cl_2_·H_2_O could promote MCF-7 cell death through activation of autophagy and have anti-metastatic and anti-angiogenic effects [71]. Furthermore, the HSA-IND-C_4_ complex delivery system constructed based on copper (II) 2-isopropylpyridone thiocarbamate compounds has been shown to induce apoptosis and autophagy in cancer cells and inhibit inflammation and angiogenesis within the tumor tissue mesenchyme, thereby remodeling the tumor tissue mesenchyme [43]. These findings show that copper complex-induced autophagy gives it a variety of anticancer activities and is a good candidate for cancer therapeutic agents

#### 3.2.2. Exhibiting Cytotoxicity and Inducing Cancer Cell Death

It is worth noting that the potential of copper complexes to selectively kill tumor cells is superior to cytotoxic drugs such as cisplatin [119]. Compared with cisplatin and oxaliplatin, [Cu_2_(μ-Cl)_2_L_2_]-CH_2_Cl_2_ showed stronger cytotoxicity against some selected tumor cell lines based on ROS-induced autophagy while exhibiting reduced toxicity against normal cells under identical conditions [70]. Similarly, copper (II) complexes with isoquinoline derivatives as ligands showed high cytotoxicity against A549 cells and low cytotoxicity against normal human cells [56]. Moreover, the Schiff base Cu (II) complex has been reported to cause a wide range of cell death types, and it appears to be more inclined to induce autophagy with antiproliferative activity against cancer cells (MCF-7) and less toxic to healthy cells (HEK-293T) [60]. Furthermore, Choroba et al. found that the Cu (II) complex [Cu(4′-(2-quin)-terpy) Cl] (PF_6_) induced autophagy activation in cancer cell line A2780 and HCT116 with strong cytotoxicity, while the cytotoxicity was lower in normal dermal fibroblasts [54]. Moreover, it was shown that copper (II) complex C2 was cytotoxic to human bladder cancer T24 cells but was much less cytotoxic to human normal liver HL-7702 cells and lung fibroblast WI-38 cells [50]. However, it has been shown that the novel optically pure dinuclear copper (II) complexes of the rosin derivative dehydroabietic acid can induce autophagy in MCF-7 cells to produce cytotoxicity comparable to that of cisplatin and oxaliplatin [69]. In addition, activation of autophagy induced by copper (II) binucleated imine complexes was capable of causing cancer cell death and was highly cytotoxic to melanoma cells, especially human SKMEL-05 cells [120]. Sequeira et al. have confirmed that the autophagy-inducing effect of Cu (I) complexes could cause HCT116 cell death, and the cytotoxicity of Cu (I) complexes on tumor cells was significantly higher than that on healthy cells [44].

Several studies have shown that copper complexes can induce autophagy and thus cause cancer cell death, or cause cell death through autophagy-induced apoptosis in cancer cells [77,83]. SBCCC has been reported to induce cancer cell death via ROS-mediated autophagy activation [63]. Similarly, it has been reported that dinuclear copper (II) complexes exhibit good anticancer activity in vitro by inducing autophagy to cause cancer cell death [67]. Moreover, it has been reported that the copper compound Cas III-ia can induce cell death through autophagy and might be a candidate for the treatment of human malignant gliomas [84]. Moreover, the [Cu(O-O) (N-N) ClO_4_]-type copper complexes could activate the autophagy process of sarcoma 180 cells to induce apoptosis and ultimately lead to the death of the tumor cells and produce antitumor activity on tumor cells in vitro [77]. Especially, studies showed that dose-dependent activation of autophagy by the anthrone–copper (I) complex caused apoptosis to promote MGC-803 cell death, resulting in an anticancer effect [110].

#### 3.2.3. Overcoming Drug Resistance

In most cases, cancer patients using metal-based anticancer drugs such as cisplatin develop resistance, leading to cancer progression or even death [121]. Recently, copper complexes have been considered as a promising metal anticancer agent that can be widely used to overcome tumor resistance to metal anticancer drugs [122]. It is well known that autophagy is associated with progression and drug resistance in various tumors [123]. For example, the copper (II) complex with 4-quinolinyl, 4-methoxy-1-naphthyl, 2-furanyl, and 2-pyridynyl substituents could induce cell death through activation of autophagy and show good antiproliferative activity against the HCT116DoxR cell line with significantly reduced cytotoxicity in normal fibroblasts (42–129× lower) detected [82]. In addition, similarly, the combination of DSF/Cu and sorafenib has been shown to improve anticancer efficacy by activating autophagy to increase the sensitivity of HCC cells to sorafenib [47]. Nevertheless, the combination of copper (I) nicotinate complex and doxorubicin (DOX) inhibited autophagy and induced cell cycle arrest, thereby enhancing the efficacy of DOX and reducing the use of DOX in HCC1806 cells [18].

#### 3.2.4. Improving the Efficacy of Chemodynamic Therapy

Chemodynamic therapy (CDT), a therapeutic approach that uses Fenton or Fenton-like reactions to generate ·OH in the tumor region, has attracted widespread attention as a novel cancer treatment strategy [124]. Nevertheless, the efficacy of CDT is not satisfactory in practice [125]. Recently, it has been demonstrated that autophagy inhibited by copper complexes can enhance the efficacy of CDT [50,53]. For instance, recent studies found that copper (II) complexes inhibited autophagy by blocking autophagic flow, thereby shutting down the self-protective pathway of oxidative stress and enhancing CDT [50]. Furthermore, the copper (II) complexes of haloquinoline Schiff base derivative-induced autophagy inhibition could enhance the effects of CDT and exhibit strong tumor suppression in the T24 xenograft model [53].

#### 3.2.5. Promoting Cancer Cell Survival

As previously described, copper complex-induced autophagy can cause cancer cell death; however, some studies also found that copper complex-induced autophagy had a protective effect on cancer cells. For instance, co-treatment with autophagy inhibitor hydroxychloroquine significantly enhanced growth inhibition of copper complex [Cu (phen) (L-tyr) Cl] · 3H_2_O, suggesting that autophagy induced by [Cu (phen) (L-tyr) Cl] · 3H_2_O promoted cell survival in MCF-7 and MDA-MB-231 breast cancer cells [79]. Moreover, the DSF metabolite diethyldithiocarbamate combined with copper (CuET) could activate autophagy and protect tumor cells from death, thereby reducing the clinical efficacy of CuET [122]. Subsequently, further research revealed that the combination of CuET with amodiaquine, which inhibits both RiBi and autophagy, significantly improved the anticancer effect of CuET [111].

### 3.3. Copper-Based NPs

Studies have shown that the nona-copper (II)-containing 18-tungsto-8-arsenate (III) exhibits anticancer activity, which is related to its induction of autophagy [87]. It has been found that the Poly/Cu nanocomplexes block both lysosomal and autophagic pathways, prompting copper nanocomplexes to selectively kill cancer cells [91]. Similarly, studies showed that the Cu (II) metal dendrimers were cytostatic and moderately cytotoxic to U937 tumor cells, and Cu (II) metal dendrimers could induce the death process via the mitochondria–lysosome axis as well as autophagic vesicle formation. In contrast, they had almost no effect on healthy monocytes [112]. Another research found that Cu_2_O-NPs selectively inhibited the growth of cancer cells and impaired the ability of cell migration, invasion, and cytoskeleton assembly in uveal melanoma [97]. Further studies have found that the mechanism might be that Cu_2_O-NPs were located in mitochondria, autophagolysosomes, and lysosomes and caused damage to them, leading to elevated ROS and over-stimulated apoptosis and autophagy [97].

Secondly, autophagy induced by copper-based NPs can counteract drug resistance generated by tumor cells, including resistance generated by chemotherapy, radiotherapy, and other emerging cancer treatment strategies. For instance, CONP-induced apoptosis by triggering ROS and activating ERK-dependent autophagy inhibited the proliferation, migration, and invasion of bladder cancer cells, inhibited the growth of bladder cancer in tumor-bearing mice, and increased the efficacy of chemotherapy with cisplatin and gemcitabine [93]. Moreover, some scholars suggested that the combination of CuO NPs (a potent autophagy inducer) with autophagy inhibitors was able to transform the MCF7 cell response into apoptosis and sensitize chemo-resistant cancer cells [113]. Therapeutic polypyridine copper complexes (AFt-Cu) encapsulated in the natural nanocarrier aprotinin have been reported to induce cell death via autophagy-dependent apoptosis and exhibit strong tumor growth inhibition when injected into the bloodstream in a multidrug-resistant colon tumor mouse model [86]. Moreover, Pan et al. constructed a NIR-II photo-triggered nanoreactor as an in situ ultrafast CuET reactor by encapsulating DSF and ultrasmall Cu_2−x_Se NPs in thermosensitive organic phase change materials (PCMs) to prepare a nanoreactor DSF/Cu_2−x_Se@PCM [114]. The antitumor activity of Cu_2−x_Se-ET was found to be similar to that of CuET, with increased autophagy being a contributing factor, owing to its unique two-dimensional-like structure [114]. These findings indicate that copper-based NPs have good anticancer activity and can counteract tumor resistance, so it has a good prospect for antitumor drug development.

## 4. Copper-Induced Autophagy and Other Human Diseases

Copper is an essential nutrient for the human body and is primarily found in the muscles, liver, and brain [126]. Menke’s disease is characterized by systemic copper deficiency, whereas WD is characterized by copper overload in tissues, particularly the liver and brain [127,128,129], suggesting that both copper deficiency and copper overload can be toxic and affect human health. WD is a condition caused by a mutation in the ATP7B gene, resulting in the accumulation of copper in liver and brain tissue [130]. Study results have proved that copper-based agents can play a therapeutic role in PD and WD via inducing autophagy [131]. Recent studies have also found that copper is also relevant in neurodegenerative diseases [132]. The brain concentrates heavy metals, including copper, for metabolism [133]. In addition, the autophagy induced by copper-based agents also has been found to play a therapeutic or toxic role in the cardiovascular system and male reproductive system [22,38]. Given that the role of copper in human health and disease has been discussed in other review articles [134,135], the relationship between copper-induced autophagy and cancer has been summarized separately in the previous paragraphs. Here, we just summarize the relationship between copper-induced autophagy and other human diseases.

### 4.1. Wilson’s Disease

WD is an inherited disorder of copper metabolism in which dysfunction of ATP7B leads to pathological accumulation of copper, particularly in the liver and brain [136]. In fact, WD also leads to the formation of Mallory body (MB) [137]. It has been shown that pathological copper overload in WD activates autophagy, thereby preventing copper-induced apoptosis and protecting hepatocytes from the toxicity of copper overload [20]. Nevertheless, we found in our cellular model that copper inhibited autophagy by suppressing ATG4B activity and thereby inhibiting autophagy [21]. This effect promoted the formation of MB, insoluble ATG4B aggregates, and p62 and ubiquitin-positive aggregates in WD [21].

### 4.2. Neurodegenerative Diseases

Previous studies have demonstrated that copper occupational exposure could increase the risk of developing PD [138]. It has been shown that chronic copper exposure induces α-synuclein accumulation and aggregation, which is associated with copper-activated autophagy [19]. Low doses of copper exacerbated copper-induced increases in α-synuclein and thus Parkinson-like changes by impairing granulomal autophagy in the A53T mouse line [139]. Moreover, copper–dopamine complexes exerted specific neurotoxic effects on neurons by inducing mitochondrial autophagy and thus caspase-3-independent apoptosis [140]. Furthermore, the results of a study suggested that the increased autophagy levels induced by CS-AT NPs could promote phagocytosis and degradation of α-synuclein PFFs by BV2 cells, a promising nano-agent for the treatment of PD [131].

### 4.3. Cardiovascular Diseases

Recent studies have implicated copper and autophagy, respectively, as protective factors in cardiomyocyte hypertrophy and atherosclerosis [141,142]. Researchers believe that copper and autophagy are potential therapeutic targets for cardiovascular diseases [142,143]. Recently, it has been shown that Cu^+^ can induce autophagy in vascular endothelial cells, suggesting the effects of copper ion-induced autophagy on the cardiovascular system [144]. In addition, nano-preparations developed with copper may also impact cardiovascular diseases by inducing autophagy. For instance, CuS-TRPV1-activated autophagy has been shown to hinder the formation of VSMC foam cells treated with oxidized low-density lipoprotein and attenuate atherosclerosis [22]. Moreover, it has been reported that CuO NPs can trigger HUVEC cell death by causing impaired autophagic flux and autophagosome accumulation, one of the mechanisms of induced toxicity of CuO NPs to the cardiovascular system [16]. Nevertheless, CuO NP-induced autophagy has been reported to promote NRF2 activation in vascular endothelial cells and mouse thoracic aorta to mitigate NP-induced vascular injury and disease [23].

### 4.4. Male Reproductive System Injuries

Guo et al. demonstrated that CuSO_4_ could induce spermatogenesis and impair male reproductive function [145]. Subsequently, further studies demonstrated that CuSO_4_ induced autophagy activation in GC-1 cells and testis [38]. On the one hand, the activation of autophagy might play a protective role through oxidative damage and inhibition of apoptosis, and on the other hand, it might also aggravate the toxicity by promoting ferroptosis, suggesting autophagy plays a positive role in attenuating CuSO_4_-induced testicular injury and impaired spermatogenesis [38]. In addition, nano-copper is toxic to reproduction, and research has found that nano-copper could promote autophagy through the AKT/mTOR pathway, leading to abnormalities and functional damage in testicular tissue [101].

### 4.5. Nonalcoholic Fatty Liver Disease (NAFLD)

It has been reported that excess copper can activate oxidative stress and autophagy, up-regulate adipogenesis and lipid metabolism, inhibit Keap1 expression, and activate Nrf2 signaling [146]. The consequence of copper-induced oxidative stress-mediated activation of the Nrf2/PPARγ pathway was lipid accumulation [146]. However, activation of autophagy alleviated copper-induced lipid deposition, thereby protecting against copper-induced NAFLD [146].

## 5. Conclusions and Future Directions

**Figure 4:**
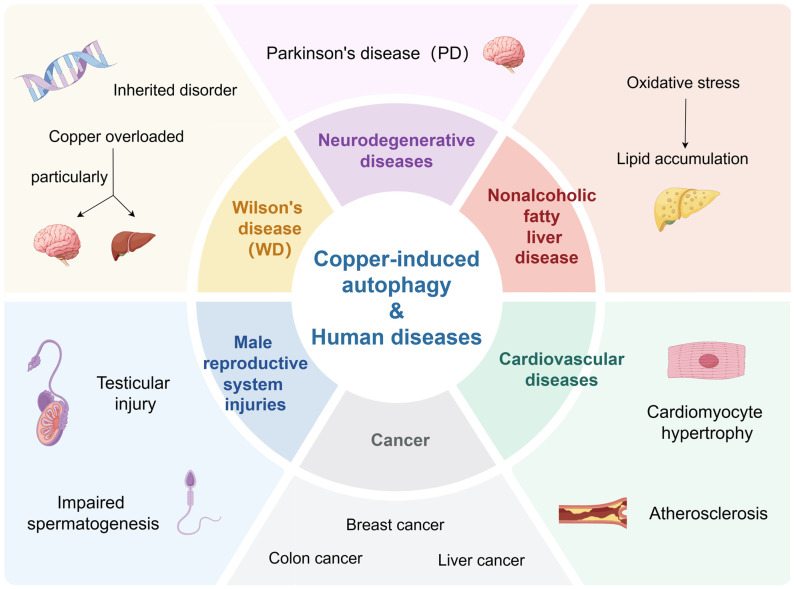
**The relationship between copper-induced autophagy and human diseases.** The role of copper-induced autophagy in human disease is complex. In neurogenerative diseases like PD and cancer, such as breast cancer, colon cancer, liver cancer, and so on, copper-induced autophagy promotes or hinders the development of diseases. In WD, a disorder of copper metabolism leads to copper overload that activates autophagy. Copper-induced autophagy reduces lipid accumulation in the liver and plays a role in preventing copper-induced NAFLD. Additionally, copper-induced autophagy not only prevents myocardial hypertrophy but also attenuates atherosclerosis. And copper-induced autophagy can attenuate impaired spermatogenesis or cause testicular injury.

Emerging studies over the past decade have convincingly shown that copper-based agents have diverse therapeutic effects on various human diseases, including cancer, PD, WD, cardiovascular diseases, and NAFLD (Figure 4). As summarized, a variety of pathways regulating autophagy are involved in the regulation of copper-based agents for treating multiple diseases. As shown in Figure 1, Figure 2 and Figure 3, copper-based agents, including copper ions, copper complexes, and copper-based NPs, mainly induce autophagy by stimulating ROS production, regulating the mTOR pathway, and sabotaging lysosomes. Overall, the autophagy-inducing effects of copper-based agents are closely related to their ability to generate excess ROS through the Fenton reaction. Interestingly, both activation or inhibition of autophagy by copper preparations can play a positive role in the treatment of human diseases. However, owing to the organ toxicity from oxidative stress, the ROS induced by copper-based preparations can possibly hinder its application. On the other hand, by regulating autophagy, copper-based agents can protect cells from death when they act as a protective mechanism, especially in cancer. The dual complexity of autophagy in disease development, and the variability in different stages of the disease endow copper-based agents with a strong potential as potent anticancer agents. Furthermore, dysregulation of the balance of redox-active biometals, such as copper, has been associated with the pathological processes of neurological disorders [147,148,149,150]. Research has shown that prolonged exposure to copper, in occupational and environmental settings, is significantly linked to an elevated risk of developing PD [139,151]. Moreover, WD was first described as a disorder of copper metabolism in 1912 [152]. In this review, we find that copper-based agents with the ability to induce autophagy may be a therapeutic drug for PD and WD, for which there is still no effective therapy.

Collectively, copper-based agents represent a promising transition metal compound for treating human diseases. However, copper-based agents are still not perfect therapeutic agents because of the regulation of inevitably induced oxidative stress. Moreover, the dual role of autophagy in disease progression is both a strength and a hindrance to further insight and application. In the future, more in-depth research on copper-based agents is needed to address some key issues, such as eliminating or reducing oxidative stress-induced organ toxicity, clarifying the role of copper-based agent-induced autophagy at different stages of the disease, designing novel copper-based agents to improve efficacy without increasing adverse effects, and addressing auto-resistance.

## Figures and Tables

**Table 1:** Study of copper ions, copper complexes, and copper nanos for cancer treatment via regulating autophagy.

Compounds	Tumor Types	Molecular Target/Mechanisms	Effect on Autophagy	Pathophysiological Effect	Refs.
Elesclomol-CuCl_2_	Prostate cancer	DLAT/mTOR pathway	Inhibiting	Enhancing the chemosensitivity of docetaxel	[17]
Cu^2+^-LFH	Human gastric cancer AGS cells	Enhanced apoptosis induction and autophagy inhibition	Inhibiting	Enhanced anticancer effects in AGS cells via PI3K-Akt-mTOR pathway activation	[39]
DSF/Cu	Human pancreatic cancer cells	Transcription factor p8 and PI3K/mTOR/p70S6K signaling pathways	Inducing	Inhibiting cell proliferation and induced apoptosis	[15]
	Colorectal cancer	ULK1	Inducing	Inducing autophagic cell death	[46]
	Human HCC cell lines HepG2, Hep3B, SNU387, and SNU423	Inducing autophagy and apoptosis	Inducing	Improving the therapeutic efficacy of sorafenib	[47]
Copper (I) nicotinate complex (CNC)	Human breast cancer cell line HCC1806	Inhibiting autophagy and inducing cell cycle arrest	Inhibiting	Enhancing the efficacy of DOX and reducing the use of DOX in HCC1806 cells	[18]
HSA-IND-C4 complex	BxPC-3 and HK-2 cells	Inducing apoptosis and autophagy	Inducing	Inhibiting tumor growth by remodeling the tumor microenvironment	[43]
Copper (II) complex C2	T24 cancer cells	Oxidizing GSH and affecting autophagy	Inhibiting	Enhancing chemodynamic therapy (CDT)	[50]
Cu(L^4^)_2_ and Cu(L^10^)_2_	T24 cancer cells	Mitochondrial dysfunctions, ER stress, and autophagy flux inhibition	Inhibiting	Enhancing the effects of CDT and exhibiting strong tumor suppression in the T24 xenograft model	[53]
[Cu(4′-(2-quin)-terpy) Cl] (PF_6_)	Cancer cell lines A2780 and HCT116	Intercalating DNA and inducing intracellular ROS	Inducing	Exhibiting cytotoxicity against cancer cells	[54]
CuL^1^Cl_2_/CuL^2^Cl_2_	A549 cells	Mitochondria-mediated apoptosis and autophagy	Inducing	Inducing bimodal death through apoptosis and autophagy	[56]
Schiff base Cu (II) complexes	Human breast cancer cells (MCF-7)	Autophagy	Inducing	Exhibiting antiproliferative activity against cancer cells but not against healthy cells	[60]
Cu (Cl_2_-L_1_) Cl	Ovarian cancer A2780 cell line	-	Inducing	Exhibiting antiproliferative activities	[61]
SBCCC	Gastric cancer cell lines SGC-7901 and BGC-823	NF-κB, ROS production, and autophagy	Inducing	Inducing cancer cell death	[63]
Cu_2_(µ2-O)(L)_4_(DMF)_2_	MCF-7	Increasing ROS, GSSG/GSH ratio, and Ca^2+^ production, etc.	Inducing	Killing MCF-7 cells and displaying anti-metastatic activities, together with anti-angiogenesis properties	[69]
[Cu_2_(μ-Cl)_2_L_2_]-CH_2_Cl_2_	MCF-7	Cell cycle arrest, apoptosis, etc.	Inducing	Exhibiting cytotoxic activity	[70]
[CuL_4_Cl]Cl·2CH_2_Cl_2_·H_2_O	MCF-7	ROS production, cell-cycle arrest, etc.	Inducing	Promoting MCF-7 cell death through activation of autophagy and possessing anti-metastatic and anti-angiogenic effects	[71]
CBP-01	Sarcoma cells	ROS augmentation	Inducing	Showing in vitro antitumor activity and cytotoxic selectivity toward the sarcoma 180 cells	[77]
[Cu (phen) (Ltyr) Cl] 3H_2_O	MCF-7 and MDA-MB-231	Inducing apoptosis and cell cycle, inducing autophagy	Inducing	Promoting cell survival	[79]
BNMPH-Cu complex	HepG2 and HCT-116	Inducing ROS generation	Inducing	Suppressing the growth of cancer cells	[80]
Copper (II) complexes 2, 3, 5, and 7	HCT116DoxR cells	ROS/affecting cell cycle	Inducing	Inducing cell death through both autophagy and apoptosis	[82]
Cas III-ia	Rat malignant glioma C6 cells	ROS and JNK	Inducing	Inducing cell death by autophagy and apoptosis	[84]
DpdtaA-Cu complex	HepG2 cells	Through p53 meditated apoptosis/ROS generation	Inducing	Exhibiting antiproliferative activities, but copper ion attenuated the antiproliferative activity of DpdtaA alone	[109]
[Cu^I^(9-AQH)_2_] · NO_3_	MGC-803 cells	Mitochondria-mediated cell apoptosis and autophagy	Inducing	Showing significant in vitro anticancer activity	[110]
CuET	U2OS osteosarcoma, A549 lung epithelial carcinoma, and MDA-MB-231 breast carcinoma	Translational arrest, p53 aggregation, and ribosome biogenesis stress	Inducing	Protecting tumor cells from death and thus reducing the clinical efficacy of CuET	[111]
Apoferritin–Cu (II) NPs	Multidrug-resistant colon tumor	Autophagy-dependent apoptosis	Inducing	Inducing autophagy-dependent apoptosis in multidrug-resistant colon cancer cells	[86]
Nona-copper(ii)-containing 18-tungsto-8-arsenate(iii)	K562 leukemia cells and HepG2 cells	Inducing cell apoptosis and autophagy	Inducing	Exhibiting antitumor activity	[87]
Poly/Cu nanocomplexes	4T1 cells	Facilitating copper ion uptake and lysosomal escaping	Inhibiting	Exhibiting synergetic effect with PD-L1 antibody through ICD-boosted T-cell infiltration	[91]
CONPs	Bladder cancer cell lines (T24, J82, 5637, and UMUC3)	Triggering ROS-induced apoptosis through activation of ERK-dependent autophagy	Inducing	Suppressing the growth of bladder cancer	[93]
Cu_2_O-NPs	Uveal melanoma cells	Elevating ROS level and over-stimulating apoptosis and autophagy	Inducing	Inhibiting cancer cell growth and impairing the ability of uveal melanoma cell migration, invasion, and the cytoskeleton assembly	[97]
Copper (II) carbosilane metallodendrimers	U937 tumor cells	Mitochondria–lysosome axis as well as autophagic vesicle formation	Inducing	Inducing death processes of U937 tumor cells	[112]
CuO NPs	MCF7	Autophagy	Inducing	CuO NP-induced autophagy is a survival strategy of MCF7 cells and inhibition of autophagy renders the cellular fate to apoptosis	[113]
DSF/Cu_2−x_Se@PCM	4T1 cells	Autophagy	Inducing	Inducing tumor cell death	[114]
